# Tricuspid Valve Regurgitation in Hypoplastic Left Heart Syndrome: Current Insights and Future Perspectives

**DOI:** 10.3390/jcdd10030111

**Published:** 2023-03-07

**Authors:** Colton J. Ross, Arshid Mir, Harold M. Burkhart, Gerhard A. Holzapfel, Chung-Hao Lee

**Affiliations:** 1 Biomechanics and Biomaterials Design Laboratory, University of Oklahoma, Norman, OK 73019, USA; 2 Department of Pediatrics, University of Oklahoma Health Sciences Center, Oklahoma City, OK 73104, USA; 3 Department of Surgery, University of Oklahoma Health Sciences Center, Oklahoma City, OK 73104, USA; 4 Institute of Biomechanics, Graz University of Technology, 8010 Graz, Austria; 5 Department of Structural Engineering, Norwegian University of Science and Technology, 7034 Trondheim, Norway

**Keywords:** engineering-based analysis, inverse finite element simulations, predictive modeling, surgical intervention, valve tissue biomechanics

## Abstract

Hypoplastic Left Heart Syndrome (HLHS) is a congenital heart defect that requires a three-stage surgical palliation to create a single ventricle system in the right side of the heart. Of patients undergoing this cardiac palliation series, 25% will develop tricuspid regurgitation (TR), which is associated with an increased mortality risk. Valvular regurgitation in this population has been extensively studied to understand indicators and mechanisms of comorbidity. In this article, we review the current state of research on TR in HLHS, including identified valvular anomalies and geometric properties as the main reasons for the poor prognosis. After this review, we present some suggestions for future TR-related studies to answer the central question: What are the predictors of TR onset during the three palliation stages? These studies involve (i) the use of engineering-based metrics to evaluate valve leaflet strains and predict tissue material properties, (ii) perform multivariate analyses to identify TR predictors, and (iii) develop predictive models, particularly using longitudinally tracked patient cohorts to foretell patient-specific trajectories. Regarded together, these ongoing and future efforts will result in the development of innovative tools that can aid in surgical timing decisions, in prophylactic surgical valve repair, and in the refinement of current intervention techniques.

## 1. Introduction

Hypoplastic Left Heart Syndrome (HLHS) is a congenital heart defect characterized by the underdevelopment of left heart components, including an underdeveloped or undeveloped left ventricle and mitral valve and/or an undersized aorta. Other possible features include a patent ductus arteriosus and a septal–atrial opening, both of which contribute to an oxygenated–deoxygenated blood mixture for systemic perfusion. Regarded together, these key features of HLHS inhibit proper blood flow and require surgical correction almost immediately after birth.

**Figure 1:**
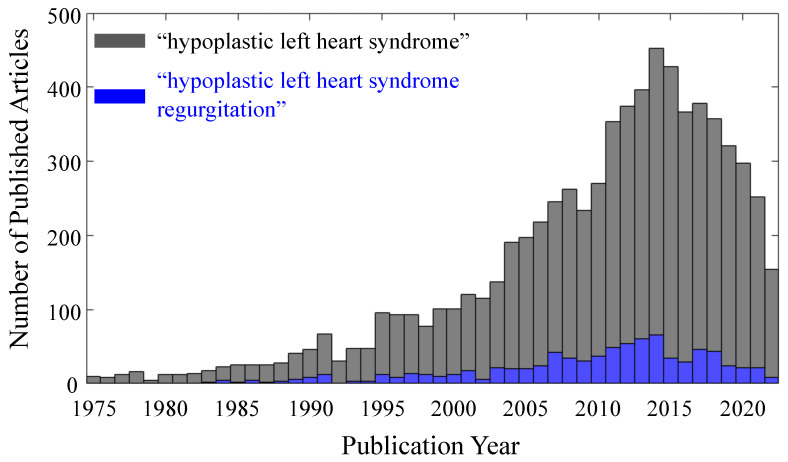
Number of citations per year with the search term ‘hypoplastic left heart syndrome (gray bars), superimposed with the subset of citations with the search term ‘hypoplastic left heart syndrome regurgitation (blue bars) (data courtesy of Lens).

This congenital heart defect has attracted increasing attention over the past two decades (Figure 1) [1,2,3]. Of those studies, there is a subset of articles dedicated to the understanding of the debilitating comorbidity of tricuspid regurgitation (TR), which is particularly dangerous in this pediatric population. There has also been great progress in distinguishing TR and non-TR cohorts and in determining the risk indicators for TR in HLHS. Looking ahead, it will prove valuable to summarize these works and identify TR indicators that have have been unanimously agreed upon, as well as those that are controversial and require further research, and to present studies that will improve our understanding of the tricuspid valve (TV) dysfunction in HLHS.

**Figure 2:**
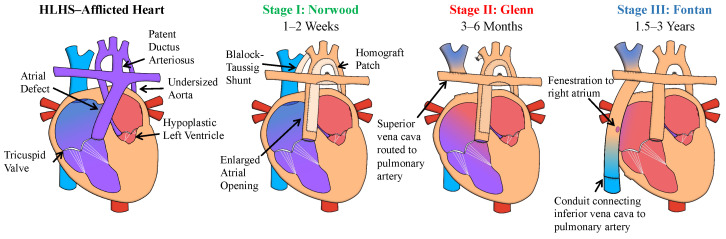
Overview of the characteristics of hypoplastic left heart syndrome (HLHS) and the timeline and surgical corrections for the three-stage cardiac palliative surgeries.

Current standard practice for the treatment of HLHS, first proposed by Norwood et al. (1980), involves three palliative (i.e., non-curative) cardiac surgeries performed within the first 1.5–3 years of the patient’s life with the overarching goal of creating a functioning single ventricular system (Figure 2) [4,5]. In Stage I Norwood surgery (or Norwood procedure), performed at from 1 to 2 weeks of age, the surgeon establishes an unobstructed blood flow between the right ventricle (RV) and the aorta, enlarges the opening of the atrial septum, and inserts a shunt to the pulmonary artery from either the aorta or the RV [4,6].

Then, Stage II (i.e., Glenn procedure) is performed at 3–6 months of age, diverting about half of the pulmonary blood flow for passive circulation by rerouting the superior vena cava directly to the pulmonary artery [7,8]. Finally, in Stage III Fontan surgery, performed at 1.5–3 years of age, the surgeon renders the pulmonary blood flow completely passive through a conduit connecting the inferior vena cava to the pulmonary artery, using a fenestration to the right atrium designed to act as a pop-off valve [9,10]. This staged palliation has resulted in a significantly improved mortality, with 87–95% of patients surviving up to 20 years or more after Stage III operation [11,12,13]. However, TR still represents a challenging risk factor and worsens the quality of life of these infants as they progress into childhood and adulthood.

Specifically, TR is present in up to 25% of all patients with HLHS undergoing staged palliation, which can result in early mortality or require additional risky, invasive TV repair surgeries (e.g., annuloplasty or valvuloplasty) [14,15,16,17]. Of the TR-afflicted patients who receive TV repair, from 22 to 48% will need a second TV repair or a total heart transplant within from 5 to 10 years after the first valve repair operation [18,19,20]. Despite the clear clinical implication of TR in HLHS, to date, there is no consensus on the guidelines or strategies to manage this comorbidity (i.e., valve regurgitation) during the three-stage palliative surgeries, possibly due to limited research to support the creation of such guidelines. Therefore, a special area of research has been devoted to understanding the indicators and mechanisms of TR in newborns with HLHS.

Given the recent advances in HLHS research, our goal in this review article is to provide a concise overview of the risks associated with TR, along with the reported indicators and mechanisms of TR. Additionally, to encourage additional studies in this clinically emerging area, we will recommend some research avenues with a focus on engineering analysis, alternative statistical methods, and predictive modeling that will help improve the current understanding of TR. Future developments such as these will be critical to facilitating personalized risk assessment and refining surgical intervention strategies in HLHS.

## 2. Tricuspid Regurgitation as a Predictor of Mortality

Historically, there have been mixed results as to whether TR was an indicator of early mortality in HLHS patients. In the period just after the advent of palliative procedure, researchers found that TR was not a significant indicator of early mortality [21,22,23]. In other studies, TR has even been considered as a contra-indicator for surgical palliation and a predestined early mortality [24,25,26]. From the perspective of inter-stage periods, Barber et al. (1988) found that TR is a significant survival factor in the inter-Stage I–II period (∼35% survival for moderate/severe TR) [14], while other researchers later contradicted this claim and did not believe that inter-stage TR is a significant risk factor [27,28,29,30]. The variations in the consideration of TR as a mortality risk extended to the post-Fontan periods.

For example, Chang et al. (1991) found TR to be an indicator for post-palliation mortality [31] and others found that TR did not significantly affect survival [32,33]. The variations in the findings may be explained by a few reasons: (i) some studies were performed shortly after the introduction of the palliative procedure, when early mortality might be due to the surgeon’s lack of experience; (ii) in some cases, the statistical analysis may have been biased (e.g., patients organized into groups that could potentially lead to a desired outcome); and (iii) patient selection bias and/or differences in hospital policies may affect study outcomes, such as Mosca et al. (2002) admitting an aggressive policy of performing valvuloplasty for all but mild or traceless TR may have skewed their findings that TR was not a mortality risk [32]. Furthermore, the widespread use of valvuoplasty in Mosca’s cohort might indicate that more aggressive treatments do not significantly affect post-Fontan TR-related mortality, and it would be beneficial to confirm this in a future study [32].

In more modern studies, pre-Norwood TR has been clearly associated with increased mortality post-Norwood: an odds ratio (OR) of 2.8[1.3–6.2] at 95% confidence interval (CI) [34]. The risk of early onset TR was also demonstrated by Sugiura et al. (2014) where they found that patients with TR in the initial palliation stage had a significantly poorer survival time than those who had developed TR in later palliative stages (42.9% vs. 92.9% at 5 years, respectively) [18]. Carlo et al. (2011) came to similar conclusions that the presence of TR on a pre-Glenn echocardiogram was associated with mortality in the interval between Stages II and III (55% attrition rate, together with a hazard ratio (HR) of 6.02[1.56–23.24] 95% CI); however, the presence of TR in the echocardiogram ‘post-Glenn was not recognized as a significant indicator of attrition (27% vs. 5% for with TR and without TR, respectively) [35]. Overall, it is clear that, with continued improvements and surgeons’ experience in performing palliative surgery, TR has been considered a significant mortality risk factor, particularly for HLHS patients who develop TR at an earlier age.

### Failure of TV Repair and Recurrence of TR

To address TR, TV repair (typically via valvuloplasty [15,32]) is performed either concomitantly with a palliative phase or during the inter-stage period. TV intervention significantly improves the 5-year survival rates (52.1% vs. 23.3% for with and without surgical repair, respectively) [18]. However, 33% of these patients suffer a *recurrent* TR within 5 years of the initial repair [18]. In addition, it was found that the patients who received TV repair concomitant with Stage I (aorta reconstruction) had sub-optimal outcomes at an 8-year follow-up compared to control (non-TR) patients: (i) transplant-free survival rates, 41% and 75% for concomitant TV repair vs. no TR, and (ii) (re-)intervention-free survival rates, 33% and 83% for concomitant TV repair vs. no TR [36]. It is clear that TV interventions for HLHS-afflicted hearts require additional research to improve the intermediate and long-term patient outcomes [37]. As a possibility, an improved understanding of the patient-specific characteristics associated with successful repairs could be beneficial for the refinement of current surgical guidelines. In particular, it was found that successful TV repair associated with the Norwood procedure is related with pre-operative anatomical and functional features: smaller dimensions of the lateral annulus, annular area, and mid-width of the RV, higher anterior leaflet excursion, and presence of anterior leaflet prolapse—the primary TR mechanism as opposed to other causes such as leaflet tethering [36]. Future studies along these lines may reveal further trends in TV apparatus related to desired repair outcomes and lead us to patient-targeted surgical guidelines, such as the suggestion of concomitant vs. inter-stage repair or a specific TV repair technique.

## 3. Identified Indicators of TR in HLHS

The mortality and risk associated with TR motivates efforts to understand the mechanisms and indicators of comorbidity, which will provide insight to guide and improve the surgical management of HLHS newborns. Additionally, it proves valuable to identify the predictors of TR onset for HLHS patient risk assessment and risk stratification. Here, we summarize some key findings from the recent literature, including geometric differences, structural abnormalities, ventricular mechanics, and surgical decisions that may be associated with TR onset.

### 3.1. Geometric Differences in TR and Non-TR Cases

Using routine echocardiography and image segmentation techniques, in-depth analyses of valvular geometry have become a promising way to understand the differences between HLHS patients with TR and without TR. For example, Nguyen et al. (2019) analyzed TV geometry in post-Fontan patients and found that patients with TR had a significantly greater change in the annulus area (16.4% vs. 9.3%), circumference (10.5% vs. 4.1%), and in septo-lateral diameter (10.7% vs. 5.7%) during the cardiac cycle compared to those without TR [38]. These changes were also found to vary regionally, with all but the septal annulus quadrant showing significant changes in these geometric quantities. A multivariate logistic regression model also revealed the mid-systolic anterior papillary muscle angle (OR =2.49[1.34–4.65] 95% CI) and the maximum septo-lateral diameter (OR = 1.73[1.12–2.68] 95% CI) as key metrics related to TR [38]. Future studies conducted in a similar manner will be beneficial to better understand the identified mechanisms of TR identified in previous work, including the structural anomalies (e.g., leaflet tethering or annular dilation), as discussed next.

### 3.2. Structural Anomalies as Mechanisms of TR

**Figure 3:**
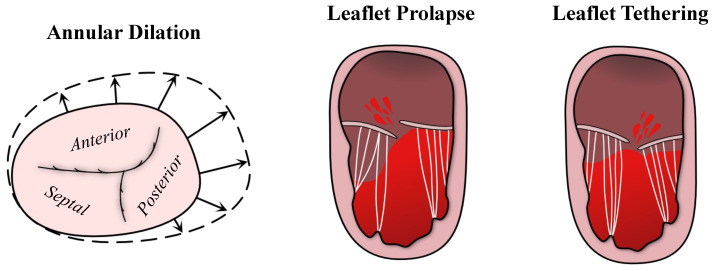
Outlining the predominant mechanisms of tricuspid regurgitation in HLHS.

The structural anomalies of the TV are primary contributors to TR in neonates and throughout the palliative series [24]. In the pre-Norwood period, the most common valvular abnormalities associated with TR include annular dilation, leaflet tethering, and chordae shortening [18,36,39,40,41] (Figure 3). Similar TR mechanisms have been identified in the inter-Stage I–II period along with other additional abnormal features such as leaflet prolapse caused by a flattened annulus and a smaller septal/anterior leaflet area [18,40,42,43]. These findings were repeated in patients analyzed after post-Glenn, with the primary TR-associated factors found as a dilated annulus (total annular area *Z*-score, 6.34±2.70; lateral dimension *Z*-score, 4.07±1.70; and antero-posterior dimension *Z*-score, 3.74±1.95), as well as anterior leaflet prolapse [44]. Other investigators also noted a narrowing of the leaflets (OR =5.8[1.2–28.9] at 95% CI) and annular dilation (OR =4.1[1.0–16.7] at 95% CI) identified as risk factors for TR after Stage II (the Glenn) palliation [45].

After the Fontan operation, the patient cohorts were analyzed to determine the from short- to long-term abnormalities associated with TR. Even before Stage I, the metrics were associated with from moderate to severe TR and a lower survival rate post-Fontan: a greater TV tethering volume (0.86±0.60 mL/m${}^{2}$ vs. 0.54±0.40 mL/m${}^{2}$: TR vs. non-TR) and annular bending angle ($155.6\pm 8.9^{\circ }$ vs. $150.3\pm 9.2^{\circ }$: TR vs. non-TR) [41]. The larger tethering volume correlated with the leaflet area (Pearson correlation factor r=0.736), annular area (r=0.651), right ventricular diastolic area (r=0.347), and change in the right ventricular fractional area (r=−0.387). In another work, the leaflet tethering was also correlated with right ventricular end-systolic areas (r=0.421), the RV sphericity index (r=0.516), and the angle between the anterior papillary muscle and the ventricular midline (r=0.316) [42]. Similar results were reported by Ono et al. (2020), noting the additional factors attributable to TR: prolapse of the anterior leaflets (52% of patients), restriction of the septal leaflets (50% of patients), annulus dilation (48% of patients), and cleft of the anterior leaflet (21% of patients) [20].

#### 3.2.1. Discrepancy in Findings with a Longitudinal Cohort

The majority of the above studies were conducted without accounting for patient-specific changes in their statistical analysis (i.e., paired samples) that might affect the identified TR-related trends and mechanisms. This is exemplified in the study by Colen et al. (2018), in which they attempted to determine compensatory adaptations of the TV during the pre-Glenn period using a longitudinally tracked cohort [46]. Although they confirmed that TR is associated with larger prolapse volumes and a more flattened and larger annulus, they found that significant TR is associated with *larger* leaflet areas. Interestingly, leaflet coaptation was not significantly different in the TR vs. non-TR groups, and they believe this to be evident for maladaptation of the TV by excessive growth—a theory they have replicated and further explored in a piglet model [42,47]. The inconsistencies in these findings may be due to the improved experimental design, which used measurements *from the same patient* pre-Norwood and pre-Glenn, in contrast to conflicting data in other works [40]. In the future, prospective studies of this type will provide better insight into the functional changes in TV during palliation periods that can lead to the development, progression, and worsening of TR.

#### 3.2.2. Summary of Primary Structural Findings

Overall, annulus dilation and leaflet prolapse or tethering are unanimously recognized as the primary mechanisms of TR in the HLHS demographic. Furthermore, the geometry of the RV also correlates with the onset of TR—the topic elaborated in Section 3.3. It should also be emphasized that the differences in the results from these studies may be due to the use of unpaired vs. paired statistical analysis designs; additional research may be beneficial to confirm whether tracking patient-specific trajectories is a more accurate method (see Section 4.2.2). In addition, discrepancies between studies may also be due to the limitations of echocardiographic technology, particularly in the previous work, as discussed in the following subsection.

#### 3.2.3. Discrepancies in Echocardiographic Findings

Echocardiographic imaging is a relatively inexpensive, non-invasive (or minimally invasive) imaging modality that is effective in diagnosing valvular disease, but there is a possibility of misdiagnoses. For example, the primary and secondary mechanisms of TR could be misidentified as demonstrated by Bharucha et al. (2013) [45]. In this work, the surgeons identified annular dilation and leaflet dysplasia as the primary TR mechanisms during surgery, while echocardiogram radiologists overwhelmingly reported only leaflet prolapse. Similar discrepancies in echocardiogram-based evaluation and interpretation can be found in the literature describing the location of the regurgitant jet. For example, using 3D echocardiography as a baseline Mah et al. (2021) demonstrated that 2D imaging had lower sensitivity and a poorer accuracy compared to 3D imaging [48]. They observed that, in pre-Glenn patients, the primary jet was in the center of the valve (70% of patients), whereas 2D echo analysis identified the antero-septal commissure as the most common (a finding similar with other 2D imaging work [44]). In a later study analyzing pre- and post-Fontan groups, they found that the center of the valve and the antero-septal commissure were equally common jet locations (45% and 48% of patients, respectively), thus revealing a temporal difference in the jet position [43,48]. Clearly, 3D echocardiography is more recommended for correct valve diagnoses, but the resolution of these images may need to be improved to reduce the inter-study discrepancies. In addition, the guidelines for echocardiogram-based clinical evaluation of TR in HLHS patients could improve diagnostic accuracy, e.g., researchers have advocated detailed temporal analyses of the vena contract and proximal isovelocity surface measurements to ensure a more accurate and reliable grading of TR severity.

### 3.3. Ventricular Mechanics and Its Relation to TR

The TV and the RV are structurally and functionally related, and the dysfunction of the RV can also produce an important contribution to the development of TR [49]. From a functional point of view, mechanical dyssynchrony and inhomogeneous contractions of the RV have been identified in the neonatal stage as mechanisms for the occurrence and recurrence of TR [45,50]. At the same time, the presence of TR can impair the RV function, as noted by Bellsham-Revell et al. (2013), who found that the presence of TR increases the indexed end-diastolic volume by 16 mL, on average [51]. RV dysfunction is also influenced by the hypoplastic condition of the left side of the heart, with one study reporting reduced basal–septal RV strains in HLHS patients with a visibly developed left ventricle [52]. There are conflicting reports on whether this hypoplasia is associated with TR: in one study, researchers identified severe underdevelopment of the left-side heart associated with TR in the post-Glenn period [53], while others found this trend in the post-Norwood period [40,52]. From a geometric perspective, greater RV (i.e., apex-to-base length, diastolic area, etc.) was associated with TR throughout the palliative series [18,36,41], i.e., the enlarged RV causes lateral papillary muscle displacement and consequently results in leaflet tethering and TR [39,40]. In conclusion, the function of the RV and TV is clearly closely related, and it is worth exploring RV repair options as a supplement or alternative to traditional TV repairs for HLHS patients undergoing staged palliative surgery.

### 3.4. Surgical Decisions and Potential Impacts on TR

During palliative procedures, the decisions made during the surgery can affect the development of TR. For example, pre-surgical planning can have a major impact on long-term mortality and outcomes; the type of shunt used in the Norwood procedure can affect TR development; and alternative procedures or modifications of each palliation stage may improve or worsen the patient’s subsequent condition. Here, we review the potential impact of these decisions at each stage of the palliation.

#### 3.4.1. Fetal Diagnoses and Pre-Surgical Planning

With recent advances in echocardiographic technology, HLHS and TR can be detected as early as fetal development [54,55,56]. Prenatal HLHS diagnosis has been associated with a lower incidence of TR [57,58]. However, it is likely that these findings are subject to patient selection bias. Despite this potential limitation, prenatal diagnosis can improve the surgical management of HLHS through prophylactic interventions after birth and pre-planned surgical treatments [57,59], or, in some cases, fetal interventions and HLHS/TR prevention strategies such as balloon dilation [60], maternal hyperoxygenation [61], or aortic valvuloplasty [62]. The preoperative management of HLHS may also affect the development of TR, with one study reporting that neonates without mechanical ventilation were at greater risk of developing TR [63].

#### 3.4.2. Norwood: Shunt Selection

During the Norwood procedure, a shunt is used to increase blood flow to the lungs, and the type of shunt used may vary based on clinician’s preference. The two main shunt types seen clinically are the Blalock–Taussig shunt (BTS) and the right ventricle-to-pulmonary artery shunt (RVPAS). The BTS connects the innominate artery or the right subclavian artery (i.e., the first branch of the artery from the aorta) to the pulmonary artery, and Asaki et al. (2014) found that the size of the BTS had a significant impact on the rate of TR prior to the second palliation stage: 3.5 mm shunt, 36% of patients; and 3.0 mm shunt, 7% of patients (OR 7.3[1.4–38] at 95% CI) [64]. Comparing BTS and RVPAS, some researchers have found that RVPAS reduces short-term inter-stage mortality and TR [65], while others found no significant differences in the short-term mortality between the shunt types [66]. However, in the long term, a meta-analysis found that the two shunt types achieved comparable survival in the period between Stages II and III and up to 6 years post-Fontan [67]. This was also evident in a post-Fontan follow-up analysis, although the BTS recipients had higher TR values [68]. Based on these clinical studies, it is possible that there are long-term complications with the RVPAS that cause this ultimate equivalent performance, such as pulmonary artery stenosis and shunt re-interventions. In addition, it is possible that the optimal shunt device or method has yet to be found.

#### 3.4.3. Alternatives to the Norwood Procedure

The hybrid procedure was proposed as an alternative to the Norwood procedure, with the overall goal of avoiding neonatal ‘open-heart surgery in infants. In this procedure, a stent is placed via a transcatheter in the patent ductus arteriosus, and the pulmonary arteries are connected in an open-chest procedure to balance the pulmonary and systemic blood flow [69,70,71]. When comparing the hybrid operation with the traditional Norwood procedure, it was found that the development of the TR does not significantly differ [72,73]. It was also found that the functional indices of RV and TV were not significantly improved in the hybrid procedure and, therefore, the benefits of adopting this hybrid procedure are not clearly demonstrated [73]. Further investigation is needed to determine whether any of the variations of the Norwood procedure (e.g., the Sano modification using the RVPAS) or other alternatives have clear long-term clinical advantages [74].

#### 3.4.4. Stage II (Glenn) Operation

During the Glenn operation, certain surgical decisions may be related to the onset of TR in the pre-Fontan period. For example, it has been observed that recipients of a TV repair at the time of the Glenn surgery have a higher risk of death [75]. Another study also examined the use of a bidirectional superior cavopulmonary anastomosis during the Glenn procedure and found that there was an insignificant difference in the development or severity of occurrence of TR [76]. However, the patients who develop TR after surgery have a higher risk of adverse late effects (OR =16.5[4.4–62.6] 95% CI) [77]. In some HLHS-specialized centers, the hemi-Fontan procedure is performed instead of the Glenn procedure, facilitating the connection of the superior vena cava to the pulmonary artery with a homograft patch, as opposed to a translocation of the artery to simplify the later Fontan operation [78]. To date, it has been established that the hemi-Fontan procedure is hardly associated with TR [79] and shows similar results to the Glenn in the short-term procedure [80]. Future long-term studies may further demonstrate the benefits or undesirable consequences of these Stage II procedures. For example, it has been theorized that the reduction in the ventricular volume loading after the Glenn procedure may lead to a reduction in TR severity, and, if the appropriate evidence is provided, this information could be useful for determining the surgical timing of Stage II (e.g., performing the operation at an earlier time if the patient has high grade TR) [81].

### 3.5. Mechanisms of Post-Repair Recurrent TR

In addition to the decisions made during the palliation stages, the method of TV repair can also influence the recurrence of TR. Annuloplasty was predominantly used in one patient cohort and it was found that the repair significantly improved leaflet coaptation lengths, reduced partial change in the RV area and diastolic area, and improved the grade of TR (reduced from 3.1 to 1.7 on a 0.0–4.0 scale) [82]. It has also been shown that preserving RV function in TV repair prevents severe TR recurrence [17,50,83]. It is worth noting that the TV repair can deteriorate the RV function immediately, but long-term improvements are observed such as reduced RV dimensions [15,17,18,82]. In addition, repair beyond an annuloplasty predicts poor prognosis (e.g., mortality) [50]. Other risk factors 5 years after TV repair were analyzed using multivariate analysis: (i) risk factors for mortality—lower weight at time of repair (HR =0.74[0.55–0.99] 95% CI); (ii) risk factors for re-operation—intervention before Stage II (HR =5.51[1.063–29.62] 95% CI) and lower weight at initial TV repair (HR =0.77[0.63–0.95] 95% CI); and (iii) risk factor for TV replacement—pre-Stage II intervention (HR =36.92[2.17–629.63] 95% CI). Unsuccessful TV repair can also be associated with the presence of posterior leaflet prolapse and septal leaflet tethering [42]. Overall, it is clear that TV repair can be beneficial in preserving RV function and that patients requiring neonatal TV repair typically have poor outcomes. In future studies, analysis of concomitant repair could be beneficial as it is believed that TV intervention prior to RV dysfunction can improve survival in HLHS patients [17,83,84].

## 4. Lacking Knowledge and Future Perspectives

Although a wide range of research has been conducted to analyze the occurrence or recurrence of TR in HLHS, there are remaining gaps in the knowledge that are critical for refining therapy and assessing patient risk. On the one hand, the list of potential TR indicators may be incomplete, and additional factors need to be considered by analyzing the tissue biomechanics of the TV apparatus. On the other hand, improved statistical designs could provide more accurate predictors of TR and the potential for individualized risk assessment models.

### 4.1. Engineering Insights for an Improved Understanding of TR

To date, the focus of TR-related research has been on the valvular geometric indicators, structural abnormalities, or problems with TV repair. These findings can also be leveraged by understanding tissue biomechanics, which could provide valuable insights into the driving mechanisms of TR. For example, excessive tissue strains or stresses, possibly caused by the change in the cardiovascular environment, could indicate the future onset of TR due to deviation from a homeostatic strain or stress that is more desirable for whole valve tissue function and valvular interstitial cells. The development of image-based engineering analysis tools and refined computational models is a crucial next step toward a better understanding of the onset of TR in the HLHS population.

#### 4.1.1. Non-Invasive Analysis of Tissue Biomechanics

Image-based analysis of valvular structures is a research area with growing potential as medical imaging techniques advance. Echocardiograms, in particular, have enabled most of the research examined, and this routine imaging modality can be used for more advanced, engineering-focused analyses. For example, in our research group, we performed image segmentation of the TV annulus in healthy and HLHS-affected newborns to gain preliminary insight into the main differences in valve function: the HLHS-affected TV annulus is larger in circumference, area, and antero-posterior diameter and becomes more circular and curved during valve closure than its healthy counterpart [85]. From an an engineering mechanics perspective, the tissue strains did not differ significantly between the cohorts, which may provide an indication that TV is remodeled in the HLHS condition to maintain the homeostatic tissue strain throughout the cardiac cycle [85]. Future research can be performed in a similar way for the TV leaflets to quantify the strains, curvatures, or other biomechanical/geometric factors. In order to enable such an exploration of these dynamic, geometrically complex structures, it can be advantageous to use fully or semi-automated image segmentation methods, such as the recently developed statistical shape modeling technique [86] or deep learning [87,88]. Through the development and refinement of these engineering-focused and automated image segmentation frameworks, the non-invasive analysis of TV becomes more manageable in the clinical setting, which can eventually lead to a detailed, individualized patient risk assessment.

#### 4.1.2. Computational Modeling

Information about tissue strains and geometry can only be obtained using the image-segmented valvular structures, but in silico computational modeling, on the other hand, can further predict hemodynamics, tissue material properties, and/or stresses of the TV apparatus in vivo. For example, computational models were developed to compare the hemodynamic environment using different shunts in the Norwood procedure [89]. Computational models and simulation frameworks in the HLHS demographic are sparse, and existent modeling techniques for the adult mitral and TV could be adapted for use with the HLHS-afflicted TV to gain new insights into the potential changes in tissue properties that underlie and predict the mechanisms of TR. As an example of how this inverse analysis may be performed, we provide a brief demonstration using our previously presented TV finite element framework [90].

**Figure 4:**
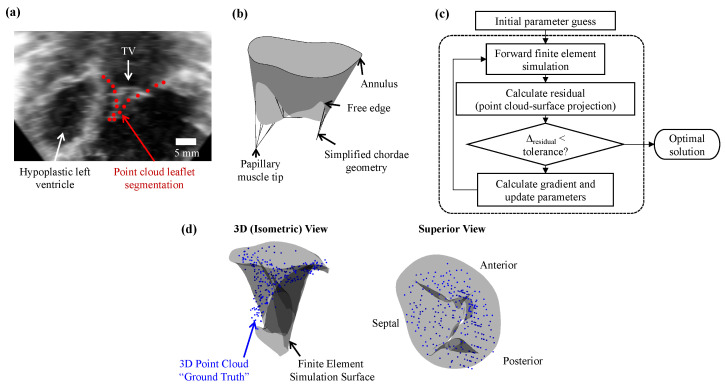
(**a**) Image segmentation of the TV leaflets from a 3D echocardiogram of a newborn with HLHS; (**b**) simplified finite element model used in (**c**) the inverse analysis framework; and (**d**) representative results from the inverse modeling.

Inverse finite element analysis requires an iterative process to match the ground-truth segmented leaflet surface or point cloud to the finite element mesh. As a proof of concept, we used 3D Slicer (an open-source image segmentation software) to manually trace the TV leaflets of a representative HLHS patient by placing individual points throughout the 3D echocardiogram image stack, obtaining the open (reference configuration) and closed valve geometry (see Figure 4a, and a simplified finite element model in Figure 4b) [91,92]. The open valve geometry was used to generate the finite element mesh for the TV leaflet surface (i.e., a lofted surface, see [93]). Then, the inverse finite element method was applied using the algorithm shown in Figure 4c, where the point cloud from the closed valve state was used to inform the deformed shape of the TV finite element surface. Herein, the residual was defined as the total projected distances of the segmented points to the finite element surface as performed in [94] and a forward difference approximation was used in the gradient-descent optimization algorithm to estimate the material parameters of the functioning TV.

Using this approach, an apparently reasonable match between the point cloud and the finite element surface can be achieved (Figure 4d), and, with refinements, these reconstructions of the TV can become a valuable clinical tool. Specifically, the simulated TV surface can be used for high-resolution visualizations, including more detailed temporal information of the valve closing than can typically be acquired in echocardiography. The predicted tissue properties from the inverse analysis can also be used to perform simulations of different treatment options or disease progression for improved surgical planning. However, it is important to note that the uniqueness of the acquired solution must be guaranteed for accurate predictions of the tissue stresses and material properties, which is a focus of our future research. Another important aspect is the realistic implementation or simplification of the boundary conditions in the simulation, including the transvalvular pressure load and the in vivo motions of the papillary muscle and the TV annulus.

### 4.2. Statistical Analysis Designs in TR-Related HLHS Research

Most of the work discussed in this article involves analyses of the differences in several recorded measurements and factors between TR and non-TR cohorts relevant to HLHS. Overall, these studies focus on answering the question: *What are the significant differences between HLHS patients with TR and without TR?* While these studies may provide new insights into indicators of TR, a more clinically useful research question exists: *What are the key predictors of TR in the HLHS population during the palliation stages?* To answer this question, another subset of statistical analysis tools must be warranted to obtain the *predictors* of the onset of TR. With more advanced statistical techniques, a predictive model could be developed to determine those patients at risk of TR based on their individual measurements in the clinic. We believe that using multivariate logistic models can lead us to answer the overarching question about the most important TR predictors [95]. To demonstrate the feasibility of this technique, we provide a brief description in the following subsections. In addition to advocating the use of multivariate models, we also briefly discuss the advantages of working with a longitudinally tracked patient cohort, which can more accurately provide the predictors of TR by accounting for patient-specific trajectories.

#### 4.2.1. Demonstration of Multivariate Logistic Modeling

**Figure 5:**
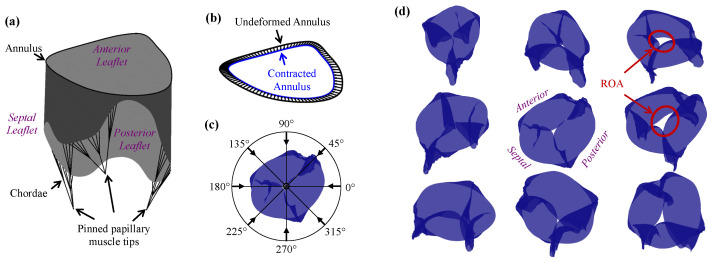
(**a**) TV model based on the average measurements of our cohort of n=8 neonates with HLHS; (**b**) visualization of the annulus contraction in the finite element simulations; (**c**) diagram depicting the top–down viewpoint (represented by the dot) and eight offset viewpoints used to (**d**) quantify the maximum regurgitation orifice area (all views from the same representative simulation).

Using our previously presented HLHS patient cohort [85] (IRB #14112 from the University of Oklahoma Health Sciences Center on 12 August 2021), we constructed a *population-averaged* TV geometry as the baseline for elucidating the mechanisms of TR. In short, we first created a collection of TV geometries covering a range of several key measurements identified as TR indicators in the previous literature: two primary annular diameters, bending angle, three leaflet heights, and area change during TV systolic closure (Table 1, Figure 5a).

**Figure 6:**
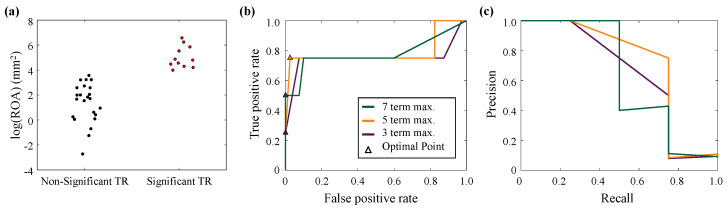
(**a**) The *k*-means clustering classification of non-TR and TR groups (note that the natural logarithm of the ROA value is used for better visualization); (**b**) ROC curves generated from multivariate logistic models using a maximum of 3, 5, or 7 predictor variables; and (**c**) precision–recall curves for each of the multivariate models.

These geometries were then used in our finite element simulation framework using the conditions and simulation specifications detailed in [90]. After the finite element simulation was completed, the simulated TV closing surface was exported and post-processed to quantify the regurgitant orifice area (ROA), which represents the TR severity (i.e., the grading of TR). In this procedure, as previously described in [96], the simulated TV surface was projected onto a series of camera viewpoints and the area of the uncoapted region was calculated, using the maximum ROA as a measure of TR severity (Figure 5b). The diagram in Figure 5c shows the top–down viewpoint and the eight offset viewpoints used to quantify the maximum regurgitation orifice area (Figure 5d). To categorize the patients with severe or non-severe TR, we used *k*-means clustering—an unsupervised classification algorithm (Figure 6a). Here, we briefly highlight that this TR quantification and classification pipeline can be useful in future work with real patient data for automated bulk data processing.

With the binary classification, we then performed a multivariate logistic regression fit using the LASSO algorithm and a 70/30 train/test cross-validation data split [97], with the determined coefficients listed in Table 2. LASSO regression can further be used to find the best *n* predictors for a multivariate model, which may be useful to determine the trade-off between model simplicity and model accuracy, as visualized by the receiver operating characteristic (ROC) curves (Figure 6b).

After developing the multivariate model, we also demonstrated the calculation of the key performance metrics, which are crucial for transparency and model assessment. On the one hand, the common performance metrics for predictive models can be summarized into four main categories: (i) *overall performance*, reflecting the differences between the predicted and actual results; (ii) *discrimination*, which describes the effectiveness of the model in distinguishing between events and non-events; (iii) *calibration*, quantifying the agreement between predicted and observed risks; and (iv) *reclassification*, the number of data points that change category with an updated form of the prediction model [98]. On the other hand, the generally adopted assessments for the multivariate logistic models include the *c* statistic (i.e., the area under the ROC curve), sensitivity SN (i.e., the true positive rate), specificity SP, and the precision PREC. The last three metrics can be determined from the number of true positive TP, false positive FP, false negative FN, and true negative TN cases based on the optimal threshold value that balances the TP and FP rates (Figure 5b), i.e.,

(1) $$SN=\frac{TP}{TP+FN},\;SP=\frac{TN}{TN+FP},\;PREC=\frac{TP}{TP+FP}.$$

For the example models developed in this review, the performance metrics revealed a trade-off between the maximum number of parameters used and the model accuracy, with at least five parameters required for the accurate prediction of the pseudo-patient condition. As an alternate measure of the model performance (particularly in the case of unbalanced observations for each group), the precision–recall (i.e., precision–sensitivity) graph can be used to visualize the model’s ability to discriminate between groups (Figure 6c).

For comparison with the multivariate model, we also performed statistical comparisons of each measurement between the two groups using Student’s *t*-test. From this we found significant differences between the two groups for the posterior leaflet height (p=0.001) and the annular area change (p=0.001). Comparing the two statistical modeling approaches, it becomes clear that the metrics that differ significantly from the *t*-tests do not necessarily indicate the measurements needed to accurately predict the onset and development of TR.

In this simple demonstration, we have shown that identifying the *predictors* of TR requires the use of a binary classification technique, such as multivariate logistic regression or support vector machines. By applying the same procedure with real patient data, insights can be obtained to clarify the key indicators of TR. In summary, the multivariate logistic model can be usefully implemented as a supplement to the statistics typically reported in the literature so far and can be helpful to identify the most important TR-related indicators.

#### 4.2.2. Longitudinal Cohorts and Mixed-Effect Models

In a more statistically rigorous study design, longitudinal statistical modeling can provide even better insight into the mechanisms of TR. As a key example of the benefits of conducting a longitudinal study, Colen et al. (2017) found that the leaflet areas *increase* in the presence of TR for their longitudinally tracked, post-Norwood cohort [46], whereas, in another non-paired sample study by Takahashi et al. (2009), TR was associated with *smaller* leaflet areas [40]. By performing a statistical analysis with repeated measurements of the same patient over time, the findings can be more representative of the underlying phenomena in TR due to the complex, multi-factorial nature of this disease condition. Of all the possible methods, mixed-effect models are the primary model of choice for many statisticians for longitudinal predictive modeling, especially on a patient-specific basis [99,100,101].

In general, mixed-effect models are a class of powerful statistical modeling techniques that incorporate both the population-averaged trends (i.e., fixed-effects) and the patient-specific variations (i.e., mixed effects). In short, these mixed-effect models have the form

(2) $$y_{i}\left(t\right)=bX+u_{i}Z_{i},$$

where $y_{i}\left(t\right)$ is the outcome of the ith patient at time *t*, and b and $u_{i}$ are the coefficients of the fixed-effect parameters X and the individual-effect parameters $Z_{i}$, respectively, [99,100]. The advantages of this statistical modeling technique include: (i) the model can still be applicable to patient dropout data (e.g., early mortality); (ii) patient-specific predictions can become more dependent on individual trends when each follow-up dataset is considered; and (iii) the time-specific probabilities of TR onset can be determined by implementation with a Cox model. We can therefore use this method in future HLHS research to optimize intervention timing. For example, a prophylactic TV repair concomitant with palliative surgery may be beneficial to proactively correct TR.

## 5. Conclusions

In this review article, we have provided a summary of the current understanding of the mechanisms and clinical implications of TR in newborns with HLHS. Primarily, TR has been found to be associated with a dilated annulus, leaflet tethering, leaflet prolapse, and right ventricular dysfunction. At the same time, the list of TR identifiers may be incomplete, and future studies focusing on engineering analysis and computational simulations will lay the foundation for a better understanding of the disease state and etiology relevant to the onset and worsening of TR. After this review, we also advocated the use of multivariate logistic models with reports on key performance indicators, which are critical for transparency and possible enrollment in the clinic. Finally, the development of a predictive model could help change the current paradigm for the management of HLHS and patient risk assessment when a longitudinally tracked patient cohort is manageable. Overall, a remarkable amount of work has been performed to understand TR in the HLHS state. Continued efforts to refine surgical timing and intervention strategies may improve TV repair outcomes and TR-related mortality rates. 

## Figures and Tables

**Table 1:** Range of geometric parameters used to construct the pseudo-population for the multivariate logistic modeling demonstration. Note that the mentioned average values are based on our preliminary observations of a cohort of n= eight neonates born with HLHS.

Geometry Parameter	Parameter Range	Description
Anterior–Posterior Diameter (mm)	[20.1,40.2]	100–200% of the average value for emulating annular dilation
Septal–Lateral Diameter (mm)	[16.5,33.1]	100–200% of the average value for emulating annular dilation
Bending Angle (deg)	[143.5,175.4]	±10% of the average value
Area Change (%)	[5, 20]	Covering the range described in Section 3.1
Septal Leaflet Height (mm)	[6.6,19.9]	±50% of the average value
Anterior Leaflet Height (mm)	[7.7,23.0]	±50% of the average value
Posterior Leaflet Height (mm)	[8.2,24.5]	±50% of the average value

**Table 2:** Coefficients determined for the multivariate logistic models considering 3, 5, or 7 terms, together with the respective area under the curve to assess the model accuracy. It should be noted that the number of parameters was chosen to obtain unique ROC curves to demonstrate the statistical technique.

Maximum Number of Parameters	3	5	7
Intercept Term	2.40	2.53	−112.63
Anterior–Posterior Diameter (mm)	0	0.05	1.33
Septal–Lateral Diameter (mm)	0.04	0.09	1.49
Bending Angle (deg)	0	0	0.77
Septal Leaflet Height (mm)	0	0	0
Anterior Leaflet Height (mm)	0	−0.06	−1.64
Posterior Leaflet Height(mm)	−0.19	−0.28	−3.08
Area Change (%)	−23.06	−31.48	−339.19
Area Under the Curve (-)	0.75	0.79	0.78
Sensitivity (-)	0.25	0.75	0.5
Specificity (-)	1.00	0.98	1.00

## Data Availability

Not applicable.
